# Determinants of Mortality among HIV Positives after Initiating Antiretroviral Therapy in Western Ethiopia: A Hospital-Based Retrospective Cohort Study

**DOI:** 10.1155/2013/491601

**Published:** 2013-02-20

**Authors:** Mitiku Teshome Hambisa, Ahmed Ali, Yadeta Dessie

**Affiliations:** ^1^Department of Public Health, College of Health and Medical Sciences, Haramaya University, P.O. Box 235, Harar, Ethiopia; ^2^Department of Epidemiology and Biostatistics, School of Public Health, Addis Ababa University, P.O. Box 25819/1000, Addis Ababa, Ethiopia

## Abstract

Studies revealed that there are various determinants of mortality among HIV positives after ART initiation. These determinants are so variable with context and dynamic across time with the advancement of cares and treatments. In this study we tried to identify determinants of mortality among HIV positives after initiating ART. A retrospective cohort study was conducted among 416 ART attendees enrolled between July 2005 to January 2012 in Nekemte Referral Hospital, Western Ethiopia. Actuarial table was used to estimate survival of patients after ART initiation and log rank test was used to compare the survival curves. Cox proportional-hazard regression was applied to determine the independent determinants of time to death. The estimated mortality was 4%, 5%, 6%, 7%, and 7% at 6, 12, 24, 36 and 48 months respectively with mortality incidence density of 1.89 deaths per 100 person years (95% CI 1.74, 3.62). Forty years and above AHR = 3.055 (95% CI 1.292, 7.223), low baseline hemoglobin level (AHR = 0.523 (95% CI .335, 0.816)), and poor ART adherence (AHR 27.848 (95% CI 8.928, 86.8)) were found to be an independent determinants of mortality. These determinants of mortality have to be taken into account to enhance better clinical outcomes of ART attendees.

## 1. Introduction 

HIV/AIDS remains one of the leading causes of death globally. It is projected to continue as a significant cause of premature mortality [1]. According to the joint 2011 HIV/AIDS report of WHO, UNAIDS, and UNICEF, an estimated 34 million people were living with HIV/AIDS globally with 2.7 million new HIV infections in 2010. Of these, 68% were residing in sub-Saharan Africa [2]. Ethiopia is one of the seriously affected countries in sub-Saharan Africa with a large number of people (approximately 800,000) that are living with HIV/AIDS and 44,751 AIDS-related deaths. An estimated number of 249,174 adults (86% of eligible) are on ART treatment [3–5]. The average life expectancy at birth is low,   51 years for males and 53 years for females. It is expected to further decline if the present HIV infection rates continue [3].

Different studies from different countries reported that WHO clinical staging, viral load, age, gender, CD4 cell count, total lymphocyte count (TLC), body mass index (BMI), ART adherence, and baseline hemoglobin level were determinants of mortality [6–10]. Even though studies had identified these determinants of mortality, they are so variable with context and dynamic across time with advancement of care and treatments as many years are being spent on highly active antiretroviral therapy (HAART) [11]. Thus, it is needed to generate locally consumable data to provide evidence for organizations working on HIV/AIDS and ART at national, regional, and district levels on factors determining the mortality of HIV positives attending ART. Therefore, the objective of this study was to identify independent determinants of mortality in PLWHA after initiation of ART and to estimate the time of death of PLWHA who are on ART. Important baseline variables like BMI, CD4 count, and TLC were included in this study to address the limitations of the previous cohort studies done in Ethiopia.

## 2. Methods 

### 2.1. Study Setting

The study was conducted at Nekemte Referral Hospital, East Wollega, from December 1, 2011, to January 1, 2012. Nekemte is an administrative capital of the East Wollega Zone. It is 331 km to the west of Addis Ababa, the capital of Ethiopia. There was one public referral hospital in Nekemte. The hospital provides preventive, curative, and rehabilitative services for populations of more than 1.5 million. It also provides patient care and treatment for HIV/AIDS. ART service program was initiated in 2005 in the hospital. A total of 6056 patients had been enrolled of which 3,200 had ever started ART and 1,711 were on ART at the time of the study [12]. 

### 2.2. Participants

The study participants were PLWHA who had been on ART in the hospital from July, 2005, to January 1, 2012, who have complete registration, intake, and followup forms. To be included, the patient has to be older than fourteen years and started ART at same hospital. Patients who get diagnosed outside of the hospital (transfer in), transfer out, loss to followup (drop, lost), women who were pregnant at the time of ART initiation, lactating mothers in WHO stage I or II who started ART exclusively to prevent vertical transmission, and patients with competing causes of death (cause of death other than HIV like accident, patients with immune-compromising chronic diseases such as diabetes, thyroid disease, or any non-AIDS malignancies) were excluded.

The study subjects were randomly selected based on the inclusion criteria. Profiles of all patients on ART between July, 2005, and January 1, 2012, were evaluated, and exposure status was first identified as stages I-II (unexposed) versus stages III-IV (exposed). Then, after loss to follow up, drop out, PMTCT, deaths with competing causes, and transfer out or patients started on ART since January 2009 or subjects with incomplete data were excluded. Finally those who fulfill inclusion criteria, unique ID number were given in increasing order for both exposed and unexposed ART groups separately. Then, simple random sampling technique was employed separately to select 416 samples from both groups (138 from stages I-II and 278 from stages III-IV) using computer-generated random number table. 

### 2.3. Data Collection

The data collection tool was developed from ART entry and followup forms being used in the ART clinic of the Hospital. The data were collected by reviewing pre-ART register, lab request, monthly cohort and follow up form, ART intake form, patients' card, and death certificate complemented by home visitors registration and phone calls done by drug adherence supporters to confirm death when patients were absent from their appointment. The most recent laboratory results before starting ART were used as a baseline value. When there was no pretreatment laboratory test, result obtained within one month of ART initiation was used. A total of three days training was given for one supervisor and two data collectors. Overall, data collection process was controlled by the principal investigator. Data quality was ensured by designing proper data collection materials, by checking the collected data daily for completeness, and thorough continuous supervision. All the completed data collection forms were examined again for completeness and consistency during data management, storage, and analysis by principal investigator. 

### 2.4. Variables

The main outcome measure was cumulative survival rates from the initiation of ART to January 01, 2012. The independent variables were sociodemographic characteristics (age, sex, religion, marital status, employment, educational status and dependent children at home), baseline clinical, laboratory and ART information (opportunistic illness, WHO clinical staging, TB test and treatment, ART treatment, chemoprophylaxis, drug allergies, hemoglobin, T-cell lymphocyte count, CD4 count, and side effects), and ART treatment.

### 2.5. Data Analysis

The data were entered into Epi-Info 3.5.3 for windows and analyzed using SPSS version 16.0. We described the patient cohort characteristics in terms of mean/median value for continuous data and percentage for categorical data. The end point in this study was death from all AIDS-related causes which was confirmed by reviewing medical registration in the Hospital, registration by ART adherence supporters, or by calling using the registered phone number. Individuals alive and on ART were censored at the end of the study period. At the end, the outcome of each subject was dichotomized in to censored or death. Finally, survival analysis and Kaplan-Meier test were used to measure the association of patient's characteristics with time from ART initiation to death. Life table was used to estimate survival after initiation of ART, and logrank test was used to compare survival curves. Cox proportional-hazard regression was used to calculate the bivariate and adjusted hazard rate to determine independent determinants of time to death. 

### 2.6. Ethical Considerations

Ethical approval was obtained from Research Ethics Committee of the School of Public Health at Addis Ababa University, College of Health Sciences for approval. Following the approval, Official letter of cooperation was written to the concerned bodies: Nekemte Referral Hospital and East Wollega Zone Health Bureau by the School of Public Health, Addis Ababa University. As the study was conducted through review of medical records, the individual patients were not subjected to any harm as long as the confidentiality is kept. To keep the confidentiality, ART clinic health officer and nurse of Nekemte Referral Hospital extracted the data from the medical records. In addition to that, no name or personal identifications were used on data collection form. The recorded data were not accessed by a third person, except the principal investigators, and was kept confidentially.

## 3. Results

### 3.1. Sociodemographic Characteristics of the Study Subjects

A total of 416 HIV-infected patients' records were reviewed for initial and repeated measurements, but repeated values are excluded from the analysis due to the incompleteness of records. Four hundred sixteen (386 alive and 30 death) adult patients were included in the study. One hundred and seventy four (41.8%) were males, and the mean age was 33.6 (SD = 9.04). Three hundred thirty (79.3%) were of age less than 40 years. Most of them (54.8%) followed Orthodox Christian. One hundred thirty five (32.5%) had primary education, and 39.4% had no formal education. Most of them (64.4%) were married (Table 1).

### 3.2. Baseline Clinical and Laboratory Information of the Cohort

The median weight at ART initiation was 51 kg (interquartile range (IQR, 45 kg–58 kg)). The mean hemoglobin level was 12.99 g/dL (IQR, 12.4–13.6). The median CD4 count was 141 cells/*μ*L (IQR, 73–199). Almost all (99.5%) were given cotrimoxzazole prophylaxis (CPT) at the time of ART initiation, and 9.9% had TB coinfection. Majorities (61.3%) were in WHO stage III, 28.4% were in WHO stage II, 5.5% were in WHO stage IV, and 4.8% in WHO stage I at ART initiation. Baseline ART regimens were d4t (30)-3TC-NVP for 242 patients (58.2%), d4t (30)-3TC-EFV for 82 patients (19.7%), AZT-3TC-EFV for 44 patients (10.6%), AZT-3TC-NVP for 37 patients (8.9%), d4t (40)-3TC NVP for 8 patients (1.9%), and d4t (40)-3TC-EFV for 3 patients (0.7%). Majority of them (95.9%) had good ART adherence, while 4.1% poorly adhered (not shown). Majority of the patients (83.7%) were initiated ART at CD4 <200 cells/*μ*L which indicates the most of them were in severe immune depression at baseline, while only small portion (5%) of the patients started ART at WHO stage IV (see Table 1).

### 3.3. Survival Analysis and Bivariate Cox Regression Analysis

**Figure 1:**
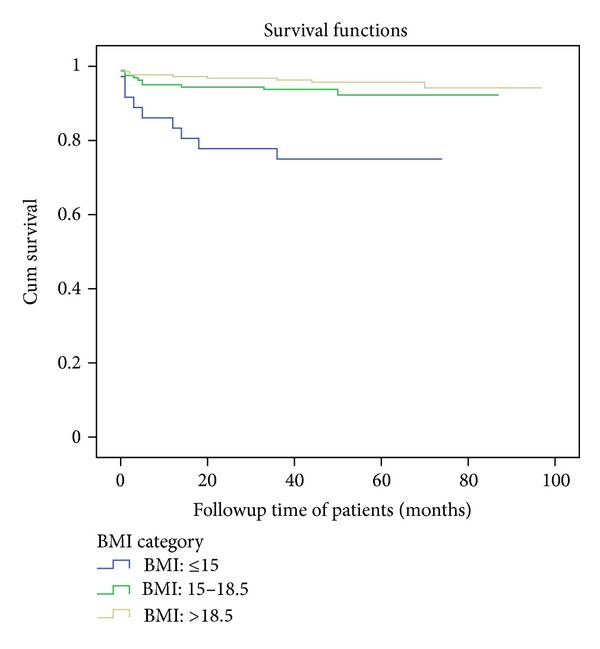
Survival functions of HIV patients by BMI category upon initiation of antiretroviral therapy between 2005 and 2012 in Nekemte Referral Hospital.

**Figure 2:**
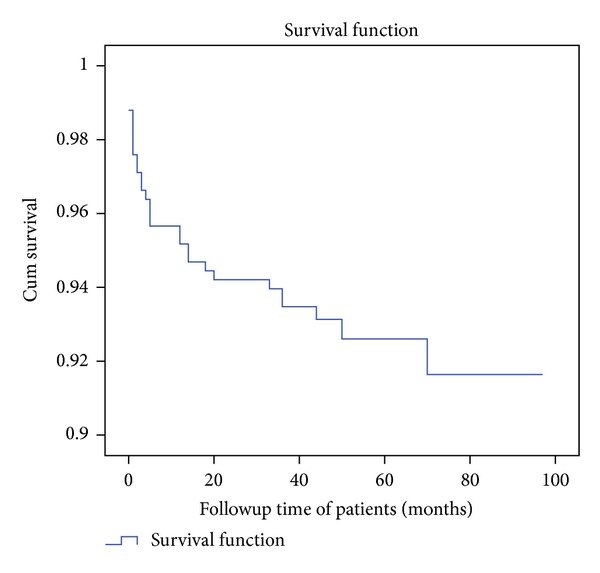
Overall survival curve of the cohort (*n* = 416) starting ART between 2005 and 2012 in Nekemte Referral Hospital, 2012.

The median followup was 47 months with minimum follow up time of 14 days and the maximum of 97 months. Thirty (7.2%) subjects were died, of which 18 (60%) were within the first 6 months. The rest (92.8%) were alive up to the end of the last censored date (January 1, 2012). The estimated mortality was 4%, 5%, 6%, 7%, and 7% at 6, 12, 24, 36, and 48 months, respectively, with mortality incidence density of 1.89 deaths per 100 person-years (95% CI 1.74, 3.62) (Figure 2, Table 2). In bivariate Cox regression analysis, age, sex, educational status, occupation, and WHO clinical staging were not associated with mortality. Functional status, dependent children at home, marital status, baseline hemoglobin, CD4 count, TLC (total lymphocyte count), BMI (body mass index), and ART adherence were all associated with mortality at *P* value less than 0.05 (Figure 1, Table 2).

### 3.4. Multivariable Analysis Using Cox-Proportional Hazard Model

In multivariable Cox regression analysis, variables that showed significance in bivariate (except marital status which was dropped as it was highly correlated with age) were entered in to the multivariable model. Age and sex of the patients were included in the multivariable model irrespective of their association with mortality as they are the most common confounding variables in epidemiology. Finally, age≥40 years AHR = 3.055 (95% CI 1.292, 7.223), lower baseline hemoglobin level AHR = 0.523 (95% CI 0.335, 0.816), and poor ART adherence AHR = 27.848 (95% CI 8.928, 86.863) were indicated as significant independent determinants of mortality. Of those, patients with poor ART adherence and older age had the highest risk of death with HR of 27.848 (95% CI 8.928, 86.863) and 3.055 (95% CI 1.292, 7.223), respectively, while one unit increase in hemoglobin level reduces HIV mortality by 48% [HR 0.523 (95% CI 0.335, 0.816)] (Table 3).

## 4. Discussions

In this cohort study, we identified 7.2% mortality rate. This was less when compared with the finding reported from Assela and Shashamane Hospitals in Ethiopia (10.3%) [8]. This lower percentage could be attributed to the fact that the real-case scenario (only confirmed dead cases) was used as events in this study which might underestimate mortality. In addition, the decline could reflect the increased care and support given to PLWHA. Most of the deaths (60%) were within the first 6 months. Similar findings were reported from different African countries including Ethiopia that attributed most of the late initiation of ART with the advanced clinical condition [9, 10, 13]. These facts were also revealed in our study in which most of the patients (84.4%) had an advanced disease as evidenced by a baseline CD4 <150 cells/mL and advanced WHO clinical stage, in which 66.8% of patients were in WHO stage III and IV. Furthermore, about half of the patients (47.6%) were malnourished at baseline (BMI < 18.5 kg/m^2^), and a considerable proportion (15.6%) of them were in severe immune depression (CD4 count <50 cells/mm^3^). 

Pertaining to serial months mortality at 6, 12, 18, 30, and 48 moths, our study found a low level of mortality when compared with study done in Zewditu Hospital in Ethiopia [14] and Malawi [15]. This survival difference might be attributed to the large proportion of the patients started ART at WHO stage I and II (33.2%), and there was a good follow up as a result of default tracers recruited by ICAP-Ethiopia program which supported the patients, high drug accessibility, and changing pattern of types of drug regimen at different time.

Previous studies reported that age of the patient was found to be the predictor of mortality, where most of the patients in older age were more likely to die [7, 16]. Similar scenario had happened in our case age 40 years and above AHR = 3.364 (95% CI 1.211, 9.348 were 3 times at higher risk of mortality than in which patients aged less than 40 years. This could be due to the fact that individuals are at higher risk of complications and respond poorly to ART as a result of combined effect of aging, HIV infection, and antiretroviral treatment [16]. It is known that as age increases immune status becomes incompetent which is consisered to be a risk for many chronic diseases which results in death. 

Patients with poor ART adherence had the highest risk of death with 27 times more likely to die than adherent patients AHR 27.848 (95% CI 8.928, 86.8). Similarly, a study conducted in Kampala, Uganda, nonadherent participants had a mortality of 42.5 deaths per 100 person-years and were two times as likely to die as adherent participants [17]. The non-adherence to HAART leads to virologic, immunologic, clinical failure, and failure to suppress viral replication, thus increasing the likelihood of developing HIV mutations that could lead to the development of drug-resistant viral strains [18]. Non-adherence to HAART also leads to failure to prevent further viral destruction of the cellular immune system with consequent reduction in the level of CD4+ cells and the development of opportunistic infections [19].  Adherence to HAART is critical to the survival of HIV/AIDS-infected people as low adherence is the main reason for poor treatment outcomes among people receiving antiretroviral therapy [18]. Other point that need attention in our study is that the hazard rate of poor ART adherence is inflated (27 times than adherent patients) and the CI is wide showing low precision and lower frequency in this category, even though it was highly significant which necessitates a large-scale study with large sample size.

Lower baseline hemoglobin level at ART initiation (moderate and/or severe anemia) is one of the independent predictor of mortality in HIV-infected patients in several previous studies conducted in the world including Ethiopia [6, 8, 9, 14]. Our study also revealed similar scenario. In this study, in contrast to other studies, in which the WHO clinical stage was found to be stronger predictor of mortality [8–10], it was not found to be associated with mortality in similar aspect of a study reported from South Western Uganda [20]. This might be due to the fact that majority of the patients (95.9%) have good ARV adherence, large proportion (33.2%) started ART early at WHO stage I and II, and very large proportion (91.4%) of patients in WHO stage III and IV were alive up to the last censored date which indicates low number of events in this category. It might also be attributed with the possibility of misclassification of WHO clinical staging as it is determined by clinical judgment of health care providers which is a common problem in developing countries with limited diagnostic capacities.

There was no significant gender difference in our study which is in agreement with the study done in Assela and Shashemane Hospitals in Ethiopia [8], though it contrasted with the study reported from Arba minch Hospital, Ethiopia, which revealed that men had a higher risk of death AOR = 1.78 (95% CI 1.47, 2.16) [7]; however, some other studies reported that females have significantly higher survival rate than males [3, 9, 21]. It had been reasoned as males were poorly adhering than females. However, in our study, there was no association between gender and ART adherence, rather majority of the patients (95.9%) have good ART adherence.

Johanssen et al. reported CD4 count <50 Cells/*μ*L at baseline, moderate and severe anemia, thrombocytopenia, and severe malnutrition as predictors of mortality [9]. Further, Ojikutu and his colleagues indicated that low BMI both in ART+ and ART− groups strongly predicts the mortality [6]. Though it can be considered as our strength to include BMI which was missed from the previous studies done in the country, base line BMI was not associated with mortality in our study which may be due to the recent nutritional intervention for severely malnourished patients in this specific hospital. 

This study has the following limitations. Selection bias is possibly introduced due to the fact that patients with incomplete records of major variables were excluded. Lack of data on viral load (HIV-RNA) during the course of ART which could have been more suitable to serve as immunological or virological marker is one limitation. Narrow scope of the study setting, study population being only from one hospital set up and population from specific area is another limitation. Missing data and limited diagnostic tests which could confirm the presence of certain opportunistic infections prevented us from analyzing the role of past opportunistic infections on survival time, though several studies report this as an important mortality determinant.

## 5. Conclusions

This study has revealed an overall lower mortality rate, but a high mortality of the cohort in the first 6 months of ART initiation; older age, low baseline hemoglobin level, and poor ART adherence were independent determinants of mortality. These determinants should be taken into account by health care providers to enhance better clinical outcomes of ART attendees.

## Figures and Tables

**Table 1:** Baseline sociodemographic characteristics of HIV patients upon initiation of antiretroviral therapy in Nekemte Referral Hospital, 2012.

Baseline variables	Frequency (*n* = 416)	Percentage
Gender		
Male	174	41.8
Female	242	58.2
Age		
Age 14–40	330	79.3
Age ≥ 40	86	20.7
Ethnicity		
Oromo	285	68.5
Amhara	105	25.2
Tigre	17	4.1
Gurage	9	2.2
Religion		
Protestant	149	35.8
Orthodox	228	54.8
Adventist	2	0.5
Catholic	2	0.5
Muslim	35	8.4
Occupation		
Farmer	59	14.2
Merchant	41	9.9
Government employee	73	17.5
NGO employee	11	2.6
Daily laborer	112	26.9
Jobless	104	25
Driver	10	2.4
Student	6	1.4
Educational status		
No education	164	39.4
Primary	135	32.5
Secondary	80	19.2
Tertiary and above	37	8.9
Dependent children		
Yes	309	74.3
No	107	25.7
Marital status		
Never married	40	9.6
Married	268	64.4
Separated	3	0.7
Divorced	29	7.0
Widowed	76	18.3
Employment status		
Working	278	66.8
Unemployed	9	2.2
Not working due to ill health	129	31.0
ARV eligibility criteria used to initiate ART		
CD4 below 200	348	83.7
WHO stage IV	21	5
WHO stage I, II, and III with TLC < 1200	47	11.3

**Table 2:** Actuarial table estimates of the cumulative progression to death for 416 study subjects starting ART between 2005 and 2012 in Nekemte Referral Hospital.

Life table				
Interval start time in months	Number entering interval	Number of deaths	Cumulative proportion of survival at the end of interval	Hazard rate
0	416	18	**0.96**	0.01
6	395	0	**0.96**	0.00
12	395	4	**0.95**	0.00
18	391	2	**0.94**	0.00
24	388	0	**0.94**	0.00
30	388	1	**0.94**	0.00
36	386	2	**0.93**	0.00
42	306	1	**0.93**	0.00
48	205	1	**0.93**	0.00
54	154	0	**0.93**	0.00
60	148	0	**0.93**	0.00
66	138	1	**0.92**	0.00
72	66	0	**0.92**	0.00
78	10	0	**0.92**	0.00
84	4	0	**0.92**	0.00
90	2	0	**0.92**	0.00

**Table 3:** Multivariate Cox regression analysis and model fit of the cohort studied (*N* = 416) between 2005 and 2012 in Nekemte Referral Hospital, Ethiopia.

Determinants	Crude HR (95% CI)	Adjusted HR (95% CI)
Gender		
Male	1.233 (0.602, 2.527 )	1.607 (0.686, 3.762)
Female	1	1
Age		
14–40	1	1
≥40	1.977 (0.925, 4.224 )	3.055 (1.292, 7.223)*
Functional status at baseline		
Working	1	1
Ambulatory	2.460 (1.129, 5.360)*	1.531 (0.676, 3.470)
Bedridden	10.634 (3.373, 33.520)*	0.891 (0.205, 3.883)
Dependent children at home		
No	4.013 (1.948, 8.266 )*	1.521 (0.651, 3.554)
Yes	1	1
Baseline hemoglobin level	0.597 (0.431, 0.827)*	0.523 (0.335, 0.816)*
CD4 count category at baseline		
CD4 count: >150	6.287 (2.318, 17.051)*	2.940 (0.750, 7.909)
CD4 count: 100–149	4.340 (1.542, 12.217)*	2.049 (0.597, 7.038)
CD4 count: 50–99	1.499 (0.423, 5.314)	0.676 (0.168, 2.724)
CD4 count: <50	1	1
Baseline TLC category		
TLC: >1200	8.183 (1.831, 36.576)*	2.656 (0.414, 17.022)
TLC: 600–1200	4.655 (1.397, 15.518)*	3.342 (0.891, 12.535)
TLC: <600	1	1
BMI category		
BMI: >18.5	6.426 (2.599, 15.890)*	1.586 (0.540, 4.662)
BMI: 15–18.5	1.553 (0.659, 3.658)	0.954 (0.361, 2.518)
BMI: ≤15	1	1
ART adherence		
Poor	52.672 (24.393, 113.735)*	27.848 (8.928, 86.863)*
Good	1	1

Note: *Statistically significant at *P* < 0.05.
